# Microfluidic Production of Polymeric Core-Shell Microspheres for the Delayed Pulsatile Release of Bovine Serum Albumin as a Model Antigen

**DOI:** 10.3390/pharmaceutics13111854

**Published:** 2021-11-03

**Authors:** Renée S. van der Kooij, Rob Steendam, Johan Zuidema, Henderik W. Frijlink, Wouter L. J. Hinrichs

**Affiliations:** 1 Department of Pharmaceutical Technology and Biopharmacy, Groningen Research Institute of Pharmacy, University of Groningen, Antonius Deusinglaan 1, 9713 AV Groningen, The Netherlands; r.s.van.der.kooij@rug.nl (R.S.v.d.K.); h.w.frijlink@rug.nl (H.W.F.); 2 InnoCore Pharmaceuticals, L.J. Zielstraweg 1, 9713 GX Groningen, The Netherlands; r.steendam@innocorepharma.com (R.S.); j.zuidema@innocorepharma.com (J.Z.)

**Keywords:** controlled release, core-shell microspheres, delayed pulsatile release, microfluidics, poly(dl-lactide-*co*-glycolide), single-injection vaccine

## Abstract

For many vaccines, multiple injections are required to confer protective immunity against targeted pathogens. These injections often consist of a primer administration followed by a booster administration of the vaccine a few weeks or months later. A single-injection vaccine formulation that provides for both administrations could greatly improve the convenience and vaccinee’s compliance. In this study, we developed parenterally injectable core-shell microspheres with a delayed pulsatile release profile that could serve as the booster in such a vaccine formulation. These microspheres contained bovine serum albumin (BSA) as the model antigen and poly(dl-lactide-*co*-glycolide) (PLGA) with various dl-lactide:glycolide monomer ratios as the shell material. Highly monodisperse particles with different particle characteristics were obtained using a microfluidic setup. All formulations exhibited a pulsatile in vitro release of BSA after an adjustable lag time. This lag time increased with the increasing lactide content of the polymer and ranged from 3 to 7 weeks. Shell thickness and bovine serum albumin loading had no effect on the release behavior, which could be ascribed to the degradation mechanism of the polymer, with bulk degradation being the main pathway. Co-injection of the core-shell microspheres together with a solution of the antigen that serves as the primer would allow for the desired biphasic release profile. Altogether, these findings show that injectable core-shell microspheres combined with a primer are a promising alternative for the current multiple-injection vaccines.

## 1. Introduction

Immunization is widely recognized as one of the greatest and most successful medical advances of the past centuries, saving two to three million lives every year by preventing or even eliminating infectious diseases [1]. However, the global coverage for many vaccines is still too low, especially in low-income countries [2,3,4]. One of the reasons for this low coverage is the limited access to routine immunization services, which is mainly a problem when multiple injections are required to obtain protective immunity against the targeted pathogens [3,4]. A multiple-injection schedule generally consists of a first immunization (primer) followed by a second or even third immunization (booster) after a certain period of time [5]. Such a prime-boost schedule does not only cause logistical problems and high costs, it is also very uncomfortable and thus jeopardizes the compliance of the vaccinee [5,6]. An example of a prime-boost vaccine is the diphtheria-tetanus-pertussis (DTP) vaccine of which, in 2020, 17.1 million infants did not receive a primer dose, and an additional 5.6 million were only partially vaccinated [2]. The latter could be prevented by developing a single-injection vaccine formulation that exhibits a pulsatile release profile and thus includes both the primer and the booster doses [5,6,7,8]. Such a pulsatile release formulation could provide for a prolonged immunological response, hence circumventing the need for multiple injections. The administration of the primer dose can easily be achieved by co-injection of a solution of the antigen or by the addition of a separate immediate-release formulation of the antigen. However, the development of the booster part of such a formulation, characterized by a pulsatile release after a predefined lag time, is challenging. Therefore, the development of a formulation providing a delayed pulsatile release is the focus of this study.

In a previous proof of concept study, the feasibility of a single-injection vaccine using a polymeric core-shell implant (oblong: ≈ 9 × 5 × 5 mm) was investigated [8]. This implant contained ovalbumin as a model antigen in a core that was surrounded by a non-porous shell of the biocompatible and biodegradable polymer poly(dl-lactide-*co*-glycolide) (PLGA). Initially, the polymeric shell formed an impermeable barrier to the in vitro release of the antigen, thereby resulting in a lag phase during which no antigen was released. Once the shell had sufficiently degraded, it lost its barrier function, which caused the antigen to diffuse out of the implant [9]. This ultimately resulted in a delayed pulsatile release profile [8]. The implant was also subcutaneously inserted in mice, and after a specified lag time, an ovalbumin-specific IgG1 antibody response was induced as expected. Moreover, it was shown that the lag time of the formulation could be tailored from 3 to 6 weeks by simply adjusting the dl-lactide:glycolide ratio of PLGA, as the monomer ratio directly influenced the degradation rate of the polymer [10,11,12]. However, such an implant has to be surgically inserted, which is obviously not ideal and therefore cannot be developed into a commercially viable vaccine product [8]. Thus, a formulation that is suitable for subcutaneous or intramuscular injection would be an interesting alternative.

To this end, we incorporated a model antigen into PLGA-based core-shell microspheres. Core-shell microspheres are vesicular particles consisting of a single core containing the therapeutic agent, which is surrounded by a polymer shell [13]. In order to prevent premature uptake by immune cells or other cells, the core-shell microspheres should be larger than approximately 20 µm but smaller than approximately 100 µm to enable parenteral administration [14,15]. Furthermore, we hypothesize that the shell thickness of the microspheres does not influence the in vitro release profile as the degradation of PLGA occurs mainly through bulk erosion [11,16]. This means that the release profile is only dependent on the polymer composition. However, there is controversy over the influence of the shell thickness, as some studies did demonstrate an increase in lag time with an increasing shell thickness [17,18,19]. In that case, a narrow particle size distribution and uniform shell thickness are necessary for obtaining a pulsatile release instead of a sustained release after the lag time. To test both hypotheses, monodisperse core-shell microspheres with shells of uniform thickness are desired. For this reason, we used droplet microfluidics as a production method, as it enables the generation of highly monodisperse particles in the micrometer range by providing great control over the size of the droplets [20,21]. In addition, the emulsion that ultimately forms the microspheres is produced drop by drop, which is in contrast to the conventional emulsion solvent evaporation method where the microspheres are produced in bulk [21]. By placing two microfluidic chips in series, a water-in-oil-in-water (W/O/W) emulsion could be produced. In the first microfluidic chip, a primary water-in-oil (W/O) emulsion of aqueous droplets containing a model antigen in an organic polymer phase was formed. In the second microfluidic chip, the W/O emulsion was encapsulated into another aqueous phase, which enabled the generation of a W/O/W double emulsion that formed the basis for the core-shell microspheres. Hence, the aim of this study was to develop core-shell microspheres containing bovine serum albumin (BSA) as a model antigen using a microfluidic setup. In previous studies, PLGA-based core-shell microspheres were produced using microfluidics but, in all cases, no therapeutic agent was incorporated [22,23,24,25,26]. To the best of our knowledge, this is the first time that microfluidics was used to produce core-shell microspheres with a PLGA shell and a core containing a (model) antigen. To assess the potential of these microspheres for application as the booster part of a single-injection vaccine formulation, in vitro release studies were conducted, and the influence of the polymer composition on the lag time was investigated by using PLGA copolymers with various monomer ratios. In a study by Sanchez et al., this relationship was already investigated using tetanus toxoid-containing core-shell microspheres, but here, both the PLGA monomer ratio and the molecular weight were varied at the same time [27]. Therefore, in this study, solely the monomer ratio was varied to determine the influence on the lag time.

## 2. Materials and Methods

### 2.1. Materials

PLGA with an inherent viscosity of 0.2 dL/g and dl-lactide:glycolide molar ratios of 50:50 (PDLG5002) and 72:25 (PDLG7502) were obtained from Corbion Purac Biomaterials (Gorinchem, The Netherlands). Polyglycerol polyricinoleate (PGPR) was a generous gift from TER Ingredients GmbH & CO. KG (Hamburg, Germany). Polyvinyl alcohol (PVA, M_w_ 9–10 kDa, 80% hydrolyzed), BSA, and fluorescein isothiocyanate (FITC) were purchased from Sigma-Aldrich Co. (St. Louis, MO, USA). Dichloromethane (DCM) and phosphate-buffered saline (PBS; 155 mM NaCl, 1.06 mM KH_2_PO_4_, 2.97 mM Na_2_HPO_4_·7H_2_O, pH 7.4) were obtained from Fisher Scientific (Leicestershire, UK). For the in vitro release medium, potassium dihydrogen phosphate, disodium hydrogen phosphate, and sodium azide were purchased from Fisher Scientific (Leicestershire, UK) and Tween 80 from Merck (Darmstadt, Germany). Trifluoroacetic acid (TFA) was obtained from VWR International Ltd. (Amsterdam, The Netherlands) and acetonitrile from Actu-All Chemicals B.V. (Oss, The Netherlands). Ultrapure water with a resistivity of 18.2 MΩ was obtained using a Millipore Milli-Q Integral 3 (A10) purification system and used for all experiments.

### 2.2. FITC-BSA Synthesis and Analysis

For the synthesis of FITC-labeled BSA (FITC–BSA), 3.2 mL of 1 mg/mL FITC in absolute ethanol was added to 20 mL of BSA solution (10 mg/mL in PBS adjusted to pH 9.4 with 1 M NaOH), after which the reaction mixture was kept under magnetic stirring for 45 min at room temperature. This resulted in a fluorophore-to-protein molar ratio of approximately 3:1. Subsequently, the mixture was dialyzed (Slide-A-Lyzer™ Dialysis Cassettes (Extra Strength), 10 K MWCO, 12–30 mL Capacity, Thermo Scientific, Waltham, MA, USA) against ultrapure water for 3 days at 8 °C to remove any uncoupled FITC. The final product was obtained by freeze drying of the resulting solution.

The labeling of BSA with FITC was assessed by thin-layer chromatography (TLC). In short, 10 μL aliquots of 1 mg/mL FITC, 10 mg/mL FITC–BSA, and 10 mg/mL BSA were applied on a TLC Silica gel 60 F_254_ plate (Merck, Darmstadt, Germany). The plate was run with a mixture of acetonitrile, DCM, and glacial acetic acid (volumetric ratio 90:10:1) as eluent and subsequently air-dried. The spots on the plate were detected at two different wavelengths (254 nm for BSA and 366 nm for FITC) with a UV lamp (Universal, CAMAG, Muttenz, Switzerland).

### 2.3. Production of Core-Shell Microspheres

**Figure 1:**
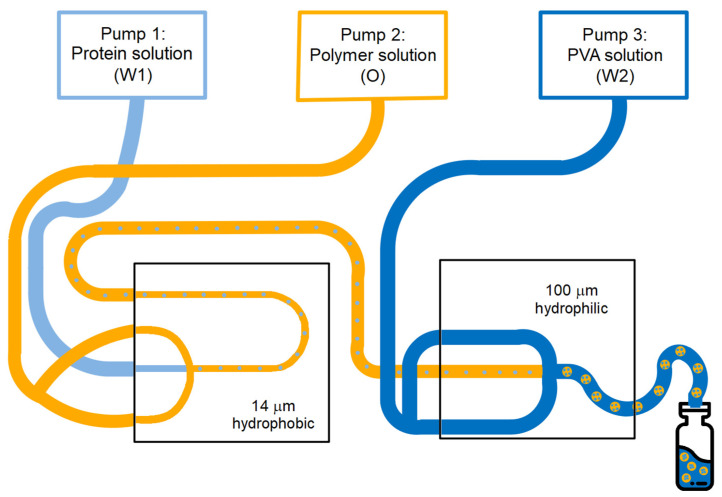
Schematic representation of the microfluidic setup used for the production of core-shell microspheres.

Monodisperse core-shell microspheres with different particle characteristics were produced by a W/O/W double emulsion solvent evaporation method using a capillary microfluidic setup (Dolomite Ltd., Royston, UK), as shown in Figure 1. For the primary water-in-oil emulsion, a microfluidic glass chip with a flow-focusing geometry, a channel diameter of 14 µm at the junction, and a hydrophobic coating was used. This coating enabled the formation of water droplets containing the model antigen dispersed in an organic polymer phase. For the secondary W/O/W emulsion, a similar glass chip was used with a channel diameter of 100 µm. This chip did not have a coating, thereby rendering the channel surface naturally hydrophilic. This hydrophilic surface enabled the formation of W/O emulsion droplets in an outer water phase, resulting in such a W/O/W double emulsion. The inner water phase (W_1_) was an aqueous 200 mg/mL BSA or 40 mg/mL FITC–BSA solution. A solution of PLGA (7.5 wt % or 10 wt %) and PGPR (0.75 wt % or 1 wt %, respectively) in DCM was used as oil phase (O). To investigate the effect of the PLGA monomer ratio on the release characteristics of the microspheres, PDLG5002, PDLG7502, and a blend of PDLG5002 and PDLG7502 (mass ratio 1:1) were evaluated. A 2 wt % aqueous solution of PVA served as the outer water phase (W_2_). All liquids were injected at independently adjustable flow rates using pressure pumps (Mitos P-Pump, Dolomite Ltd., Royston, UK). Flow and pressure were monitored using flow rate sensors (Mitos Flow Rate Sensor, Dolomite Ltd., Royston, UK). Various flow rates were used for the injection of the different phases (Table 1) in order to adjust the droplet size and thus the particle dimensions. In the first chip, the inner water phase was hydrodynamically focused by the oil phase, resulting in the continuous production of W/O emulsion droplets at the junction of the microchannels. In the second chip, the oil phase containing the inner water droplets was hydrodynamically flow focused by the outer water phase, thereby generating a W/O/W double emulsion. In both emulsification steps, the controlled break-up of the dispersed phase jet immediately at the junction of the chip ensured the formation of highly monodisperse single- and double-emulsion droplets. The obtained double-emulsion droplets were collected in an excess of PVA solution at room temperature to extract and evaporate the DCM overnight by magnetic stirring. As a result, solid microspheres were obtained that were washed three times with 0.05 wt % Tween 80 solution and three times with ultrapure water. Then, the washed microspheres were freeze-dried using a Christ Alpha 2–4 LSC plus freeze-dryer (Martin Christ Gefriertrocknungsanlagen GmbH, Osterode am Harz, Germany) of which the shelf was pre-cooled to a temperature of −45 °C. Subsequently, the pressure was gradually reduced to 2 mBar, after which the particles were dried for 3 h at a shelf temperature of −10 °C and then for 8 h at 20 °C. During this primary drying phase, ice is removed by sublimation. Eventually, the pressure was further reduced to 1 mBar during 2 h, which was followed by a final drying step of 2 h at approximately 0.05 mBar and 20 °C. During this secondary drying phase, unfrozen water molecules are removed by desorption. The settings of several process and formulation parameters were altered to obtain core-shell microspheres with varying dimensions and BSA loading. The experimental parameters and settings are summarized in Table 1. The theoretical BSA loading was calculated using Equation (1).

(1) $$\mathrm{Theoretical}\;\mathrm{loading}\;=\frac{W_{1}\;\mathrm{flow}\;\mathrm{rate}\;\times {\;W}_{1}\;\mathrm{conc}.\;}{W_{1}\;\mathrm{flow}\;\mathrm{rate}\;\times {\;W}_{1}\;\mathrm{conc}.+\;O\;\mathrm{flow}\;\mathrm{rate}\;\times \;O\;\mathrm{conc}.}\times 100\%$$

where the W_1_ flow rate is the flow rate of the inner water phase; W_1_ conc. is the mass concentration of the inner water phase; the O flow rate is the flow rate of the oil phase; and O conc. is the mass concentration of the oil phase.

### 2.4. Characterization of Particle Size and Morphology

All microsphere batches were examined before washing and freeze drying with an ME.2665 Euromex optical microscope (Arnhem, The Netherlands), and images were taken at 100×, 200×, and 400× magnification. Images of the dried microspheres were acquired with a NeoScope JCM-5000 scanning electron microscope (SEM; JEOL Ltd., Tokyo, Japan) under high vacuum at an acceleration voltage of 10 kV. For all recordings, the probe current was set to standard, and the filament setting was set to long life. The particles were mounted onto metal stubs using double-sided adhesive carbon tape and sputter-coated with gold prior to examination. The surface morphology of the microspheres was investigated at different magnifications ranging from 50× to 1500×. For each batch, ImageJ software (National Institutes of Health, Bethesda, MD, USA) was used to measure the diameter (d_y_) of the whole particle and the core of fifty randomly selected particles from several representative optical microscopy images. The volume median diameter (d50, Equation (2)) ± the standard deviation (SD, Equation (3)) and the coefficient of variation (CV, Equation (4)) of the whole microspheres and the cores were calculated to determine the particle size and particle size distribution of the different microsphere batches (Table 2). Average shell thickness was also calculated using Equation (5). The d50 ± SD and CV of the dried microspheres were determined as well, but as the differences from the wet microspheres were minimal, these values were not listed. The internal morphology was examined by first embedding the freeze-dried particles in an organic solvent-free adhesive (UHU^®^ Twist & Glue Renature, Bühl, Germany). Then, the samples were air-dried for 2 days, subsequently cooled for 30 min at −70 °C, and cut into five equal pieces using a razor blade. Finally, the cross-sectioned microspheres were examined with SEM.

(2) $$d50=\sum {V\%}_{y}\times {\;d}_{y}$$

where ${V\%}_{y}=\frac{V_{y}}{V_{\mathrm{total}}};{\;V}_{y}=\frac{4}{3}\times \;\pi \;\times {\left(\frac{d_{y}}{2}\right)}^{3}{\;\mathrm{and}\;V}_{\mathrm{total}}=\sum V_{y}$. where V_y_ is the volume of the measured particle; V_total_ is the total volume of the measured particles; and V%_y_ is the percentage V_y_ of V_total_.

(3) $$\mathrm{SD}\;=\sqrt{\frac{\sum 100\times {\;V\%}_{y}\times {\left(d_{y}-d50\right)}^{2}}{N}}$$

where N is the total number of particles measured; N = 100%.

(4) $$\mathrm{CV}=\frac{\mathrm{SD}}{d50}\times 100\%$$

(5) $$\mathrm{Shell}\;\mathrm{thickness}\;=\frac{\sum \frac{d_{y,\mathrm{particle}}-d_{y,\mathrm{core}}}{2}}{N}$$

where N = 50.

### 2.5. FITC-BSA Localization Analysis

FITC–BSA was incorporated into the microspheres at a theoretical loading of 1 wt %, as described in Section 2.3, to determine the localization of the protein in the microspheres. To this end, FITC–BSA was dissolved in ultrapure water at a concentration of 40 mg/mL, and the obtained solution was used as the inner water phase. The obtained microspheres were examined after freeze drying on a glass slide using a Leica TCS SP8 confocal laser scanning microscope (CLSM, Leica Microsystems GmbH, Wetzlar, Germany). Both fluorescence and transmitted light images were obtained using a plan-apochromat CS2 63x oil-immersion objective with 1.4 numerical aperture. FITC was excited with a 488 nm argon laser, and green fluorescence emission was collected with a 489–549 nm band-pass filter. The pinhole diameter was set at 0.7 AU (67.3 μm). To determine the protein distribution at the center of the microspheres, multiple optical cross-sections were collected at different points along the *z*-axis.

### 2.6. BSA Loading Assay

The actual BSA loading of the microspheres was determined by measuring the total nitrogen content of the microspheres using a Vario MICRO Cube elemental analyzer (Elementar, Ronkonkoma, NY, USA) in CHNS mode. The analysis was carried out at a combustion temperature of 1150 °C. The actual BSA loading was used to calculate the encapsulation efficiency (EE) according to Equation (6).

(6) $$\mathrm{EE}=\frac{\mathrm{Actual}\;\mathrm{loading}}{\mathrm{Theoretical}\;\mathrm{loading}}\times 100\%$$

### 2.7. BSA In Vitro Release Assay

All BSA-loaded core-shell microsphere batches were analyzed for their in vitro release profiles by suspending 20 mg particles in 2 mL vials containing 1 mL of 100 mM phosphate buffer (pH 7.4) supplemented with 0.05 *v/v*% Tween 80 and 0.02 wt % sodium azide. The vials were placed on a roller mixer (40 rpm) in an oven to maintain the release medium at 37 °C. At predetermined time points, the vials were centrifuged for 5 min at 1500 rpm, and 0.5 mL of the supernatant was taken and replaced with 0.5 mL of fresh release medium to keep the volume constant. The concentration of BSA in the release samples was determined by reverse phase ultra-performance liquid chromatography (RP-UPLC) with an ACQUITY UPLC Protein BEH C4 column (300 Å, 2.1 × 50 mm, 1.7 µm particle size, Waters, Milford, MA, USA) and fluorescence detection at λ_ex_ = 276 nm and λ_em_ = 345 nm. The mobile phase was a mixture of ultrapure water with 0.1 *v/v*% TFA and acetonitrile with 0.1 *v/v*% TFA in the volumetric ratio of 75:25 from t = 0–1 min and t = 1.1–2 min, and 50:50 from t = 1–1.1 min. The liquid flow rate of this mobile phase was 0.8 mL/min. The peak areas were integrated at a retention time of 1.1 min for the quantification of BSA. BSA concentrations were calculated using an 8-point calibration curve. Of some microsphere formulations, optical microscopy and SEM images were taken (see Section 2.4) both before and after 2 h, 14 days, and 25 days of in vitro release. For SEM examination of the microspheres during in vitro release, the particles were first washed and freeze-dried as described in Section 2.3. For the optical microscopy image at t = 0 days, washed and freeze-dried microspheres were suspended in in vitro release medium, after which they were immediately examined under the microscope.

### 2.8. Statistics

All core-shell microsphere formulations (A to G, Table 1) were produced once (*n* = 1). All measurements were performed in triplicate (*n* = 3), and data were expressed as mean ± SD, unless otherwise stated.

## 3. Results and Discussion

### 3.1. Production and Characterization of Monodisperse BSA-Loaded Core-Shell Microspheres

Several BSA-loaded core-shell microsphere batches with a PLGA shell and varying particle characteristics, such as particle dimensions, BSA loading, and PLGA monomer ratio, were produced using microfluidics. This allowed for the generation of particles in a highly controlled manner as the emulsion was produced drop by drop instead of in bulk. As a result, all formulations had a very narrow particle size distribution with CV values of < 10% (Table 2). The variation in core diameter was somewhat larger, although the CV values were generally still less than 10%. The average particle size of the different formulations ranged from 37.1 ± 2.8 to 48.2 ± 1.8 µm, which makes the microspheres ideal for parenteral administration through a small-gauge hypodermic needle and prevents premature endocytosis by immune cells and other cells [14,15].

**Figure 2:**
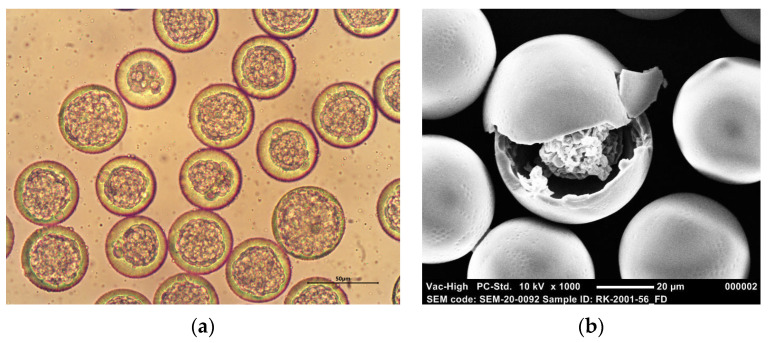
Representative images of BSA-loaded core-shell microspheres: (**a**) Optical microscopy image at 400× magnification; (**b**) Scanning electron microscopy (SEM) image at 1000× magnification. The shell of the microspheres was composed of PDLG7502, and the actual BSA loading was 5.7 wt % (Formulation F).

Furthermore, all microspheres were highly spherical, had a smooth and non-porous surface, and presented a distinct core-shell structure. Representative optical microscopy and SEM images of BSA-loaded core-shell microspheres composed of PDLG7502 are depicted in Figure 2. Before freeze drying, the cores of the particles are composed of multiple small inner water droplets (Figure 2a), as the encapsulation of one large inner water droplet posed a problem. Small fluctuations in flow are inevitable, which makes it difficult to encapsulate exactly one inner water droplet in an outer droplet. The impact of these fluctuations on the internal morphology of the microspheres will be smaller for particles with multiple inner water droplets, as these fluctuations will only alter the core diameter slightly. The inner water droplets became close-packed upon collection in PVA solution, thereby forming a distinct core, although this core still consisted of multiple separate droplets. Water was removed from the cores by freeze drying, yielding hollow single-core particles containing BSA, as shown in Figure 2b, which presumably shows the presence of BSA inside the core of a fractured particle. This indicates that the inner water droplets coalesced upon freeze drying.

The EE of the model antigen was consistently high with typical values of 80–100%, except for one formulation that had a significantly lower EE of only 23.01% (Table 2). These microspheres had slightly thicker shells than the other formulations due to the lower inner phase flow rate that was used. These thicker shells probably caused the particles to solidify slower, giving BSA the possibility to diffuse out of the cores. However, the EE seemed to be unaffected by the polymer composition, polymer concentration, BSA loading, and particle size.

**Figure 3:**
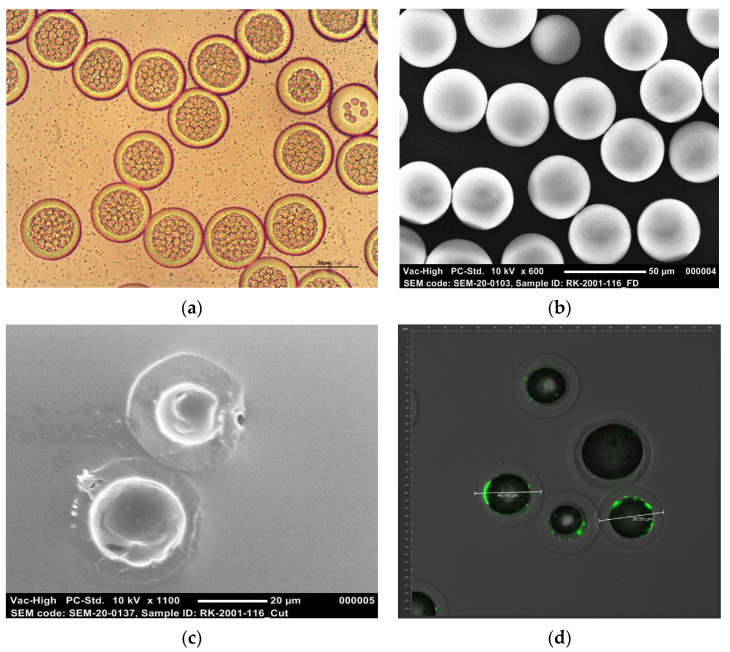
Representative microscopy images of PDLG5002-based core-shell microspheres loaded with 0.9 wt % fluorescein isothiocyanate (FITC) labeled–BSA (Formulation G): (**a**) Optical microscopy image at 400× magnification; (**b**) SEM image at 600× magnification; (**c**) SEM image of cross-sectioned microspheres at 1100× magnification; (**d**) overlay of an optical microscopy image and a confocal laser scanning microscopy (CLSM) image showing the distribution of the green fluorescent FITC–BSA.

Moreover, formulation G, which contained FITC–BSA, was produced to further elucidate the spatial distribution of BSA within the core-shell microspheres. The FITC–BSA loading was only 0.9 wt %, as the concentration and flow rate of the inner water phase were lower than for the other formulations. However, the EE was as high as 87.0%, and both the particles and the cores showed high monodispersity, which indicates that the coupling of FITC to the model antigen did not have any impact on the particle characteristics. The internal structure of the microspheres containing FITC–BSA is demonstrated in Figure 3a, and the surface morphology is demonstrated in Figure 3b. A core-shell structure is clearly visible, although before freeze drying, the separate inner water droplets are still to be seen as well (Figure 3a). A SEM image of cross-sectioned microspheres (Figure 3c) shows that the particles had obtained a single-core structure after freeze drying. The cores are virtually hollow, but some FITC–BSA seems to be present on the inner surface of the microsphere shells. This assumption is confirmed by a CLSM image (Figure 3d) that shows that the green fluorescent FITC–BSA tended to be concentrated near the inner surface of the shells and that the inner part of the cores is completely protein-free. This could be attributed to the hydrophobicity of FITC, which caused the labeled model antigen to migrate toward the polymer layer. Due to the relatively low FITC–BSA loading (0.9 wt %), only the periphery of the core was filled with the labeled model antigen. However, its fluorescence was clearly confined to the core area, and the shells of the microspheres appear to be completely free of the labeled model antigen. This indicates no or only limited diffusion of FITC–BSA into the polymer phase during microsphere formation and, thus, a clear distinction between the polymer phase and the protein phase. In turn, this might enable a delayed pulsatile release profile. A movie that visualizes the 3D structure of FITC–BSA fluorescence in core-shell microspheres can be found in the Supplementary Information (Movie S1).

### 3.2. Effect of Production Process and Formulation Parameters on Particle Characteristics

Different inner phase flow rates were used for the production of BSA-loaded core-shell microspheres to obtain microsphere formulations with varying shell thicknesses and BSA loadings. As expected, an increased inner phase flow rate generally resulted in an increased BSA loading and a decreased shell thickness (Table 1 and Table 2). In addition, the particle size somewhat increased upon increasing the inner phase flow rate. The PLGA monomer ratio was varied as well to determine its influence on the in vitro release profile. In the case of PDLG7502, the polymer concentration was reduced to 7.5 wt % to enable the production of core-shell microspheres as with 10 wt %, no primary emulsion droplets could be formed in the first chip. Therefore, the inner and outer phase flow rate were reduced as well to obtain microspheres with a similar BSA loading and shell thickness as the PDLG5002-based microspheres. Changing the polymer composition did not seem to affect the particle characteristics, as the EE was still sufficiently high, and the d50 of the particles was within the desired size range.

### 3.3. Effect of BSA Loading and Shell Thickness on the In Vitro Release of BSA from PDLG5002-Based Core-Shell Microspheres

**Figure 4:**
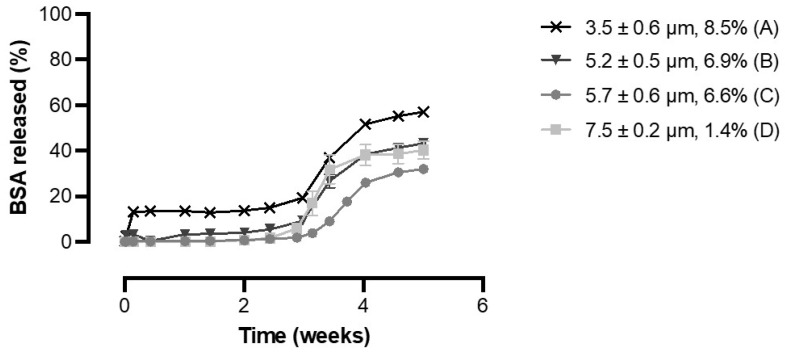
Cumulative in vitro release of BSA from PDLG5002-based core-shell microspheres with different shell thicknesses and BSA loadings (*n* = 3).

To determine whether the shell thickness is a key determinant of the lag time, PDLG5002-based core-shell microspheres with a narrow particle size distribution but different shell thicknesses were produced (Table 2). The shell thickness was tuned from 3.5 to 7.5 µm by varying the inner phase flow rate. Figure 4 shows the influence of the shell thickness on the BSA in vitro release profiles for these formulations. All formulations exhibited a delayed release profile with a lag phase of 3 weeks followed by a clear increase in BSA release over 1 to 2 weeks. A limited initial burst release was found for formulation A, but for all formulations, no additional BSA release was observed during the lag phase. The observed lag time is in line with previous studies where drug was released from core-shell microspheres [28,29] and implants [8,30] with a PDLG5002 shell after a lag time of 3 to 4 weeks. Thus, it can be concluded that the lag time does not depend on the shell thickness, at least for core-shell microspheres within the investigated size range and perhaps even for formulations with a much thicker shell, such as the abovementioned core-shell implants [8,30]. These implants had a shell thickness of approximately 1.5 mm, which is 200 to 450 times the shell thickness of the core-shell microspheres developed in this study. A possible explanation for this finding is that PLGA is a bulk-degrading polymer and not surface eroding [11,16]. Consequently, water is able to permeate through the PLGA shell, resulting in swelling and eventually bulk degradation [31,32,33]. Initially, the non-porous shell serves as a barrier to drug release, thereby causing a lag phase during which no BSA is released. However, water penetration can directly occur throughout the whole polymer layer, but this uptake of water does not lead to such swelling that BSA directly diffuses out of the microspheres [32,33]. Upon water penetration, bulk degradation of the polymer starts, and when the degradation of the shell reaches a critical level, it can no longer serve as a barrier. This causes BSA to diffuse out of the microspheres. Consequently, the lag time solely depends on the polymer characteristics and not on the thickness of the shell, which is in accordance with our hypothesis. However, other studies have demonstrated a clear relationship between the shell thickness and the onset of the pulse [17,18,19]. In those studies, the lag time ranged from 3 to even 5 weeks. The pulse occurred at the time that the shell of the microspheres ruptured, which was also shown by SEM [17]. It is unclear why different results were obtained.

BSA loading also seemed to have no effect on the in vitro release profile (Figure 4). The non-porous shell entirely prevented the release of BSA during the lag phase, and once the shell had sufficiently degraded, a large part of the encapsulated BSA was released at once, independent of the BSA loading. The formulation with the highest BSA loading did show a minimal burst release of 14.0 ± 1.6%, but this could rather be attributed to BSA release from some fractured particles with thin shells that were visible on SEM images (data not shown) than to the BSA loading.

However, for all formulations, the percentage of the total BSA content that was released during the pulse was only 30 to 50%. Incomplete release of proteins is a common problem for PLGA-based drug delivery formulations, even for relatively stable model antigens such as BSA, and it is often ascribed to protein instability within the formulation [8,34,35]. Possible explanations for protein instability are the polymer degradation products that are formed upon hydrolysis of the polymer, which can both create an acidic microclimate within the formulation and can cause protein aggregation due to the incompatibility of the protein with the polymer degradation products [36,37,38]. In addition, adsorption of the protein to the hydrophobic polymer surface can cause part of the protein to remain entrapped [36,37,38]. Therefore, further research into alternative polymers that generate less or no acidic degradation products while maintaining a delayed pulsatile release profile is desired. However, the BSA release does seem to continue after the pulse, although at a lower rate. This is caused by ongoing hydrolysis of the polymer, leading to a second phase of release in which BSA slowly diffuses out [11]. Nonetheless, it is not expected that this phase will ultimately lead to complete release of the encapsulated protein.

### 3.4. Particle Morphology of PDLG5002-Based Core-Shell Microspheres during BSA In Vitro Release

**Figure 5:**
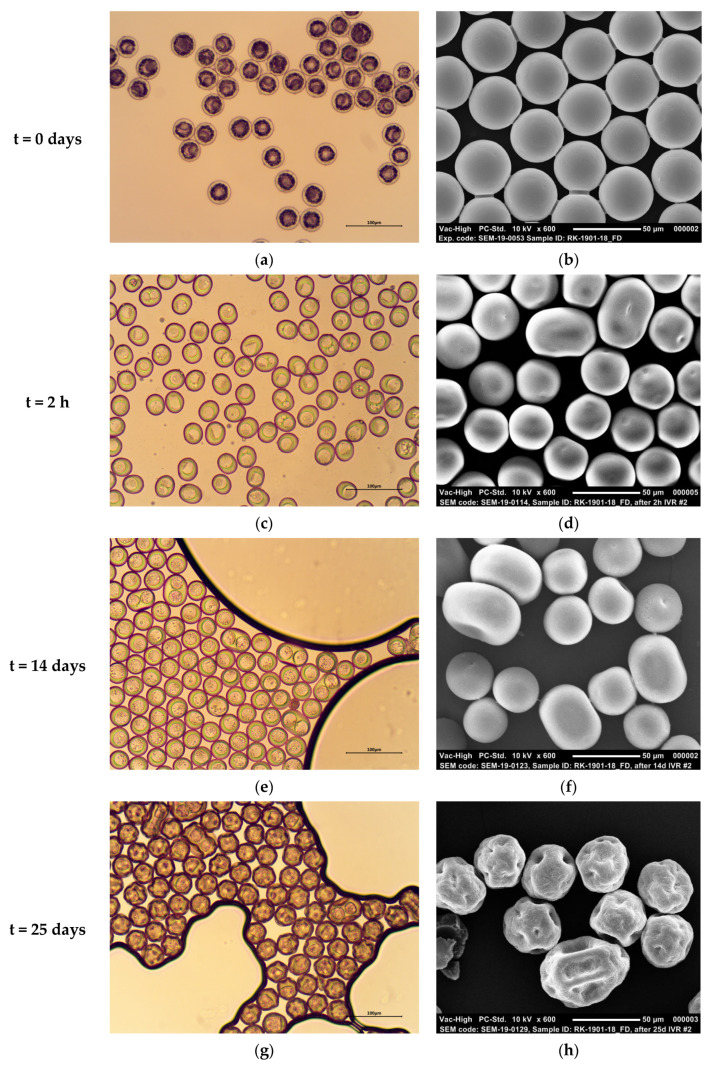
Representative microscopy images depicting the morphology of PDLG5002-based core-shell microspheres (formulation C) at different stages before and during the in vitro release of BSA. (**a**,**b**) Images of the initial microspheres after washing and freeze drying and before release; (**c**,**d**) 2 h after release; (**e**,**f**) 14 days after release; (**g**,**h**) 25 days after release. Left panel: optical microscopy images, right panel: SEM images.

To further clarify the BSA in vitro release mechanism, PDLG5002-based core-shell microspheres containing BSA (Formulation C) were imaged by optical microscopy and SEM at different time points during the in vitro release study (Figure 5). Before incubation in in vitro release medium, highly monodisperse core-shell microspheres with thin shells and a single core are visible (Figure 5a,b). The cores seem free of water due to the freeze drying, but the imprint of the inner water droplets can still be seen in the shells, and in some microspheres, an accumulation of BSA is visible in the cores. Upon incubation in in vitro release medium at 37 °C, the microspheres retained a smooth surface for at least 14 days, although the sphericity of the particles reduced (Figure 5e,f). In addition, after 2 h, some agglomeration had already occurred (Figure 5c,d). Moreover, water seems to have penetrated into the cores, as BSA is not visible anymore, and the imprint of the small inner water droplets has disappeared. This can be attributed to the glass transition temperature of the polymer, which was 31.6 °C as dry product and 19.1 °C after adding a small volume of water to the sample and allowing it to moisturize for 30 min [7]. Subsequently, the excess of water was removed, and the sample was measured with differential scanning calorimetry 5 h later. The measured glass transition temperature is below environmental temperature when set at 37 °C, which causes the polymer to change from the glassy state into the rubbery state [7]. This increases the mobility of the polymer chains, thereby enabling water influx into the cores [39,40]. Additionally, the polymer heats up and absorbs water, which is reflected in the swollen shells [11]. This transition into the rubbery state might also have caused the agglomeration of some of the microspheres. After 25 days, the microspheres had collapsed and presented a raisin-like structure (Figure 5g,h). These results are in accordance with the in vitro release profile (Figure 4) that demonstrated a clear increase in BSA release from week 3 to 5. At this point, polymer degradation has reached a critical level, which caused BSA to diffuse out of the microspheres.

### 3.5. Effect of PLGA Monomer Ratio on the In Vitro Release of BSA from PLGA-Based Core-Shell Microspheres

**Figure 6:**
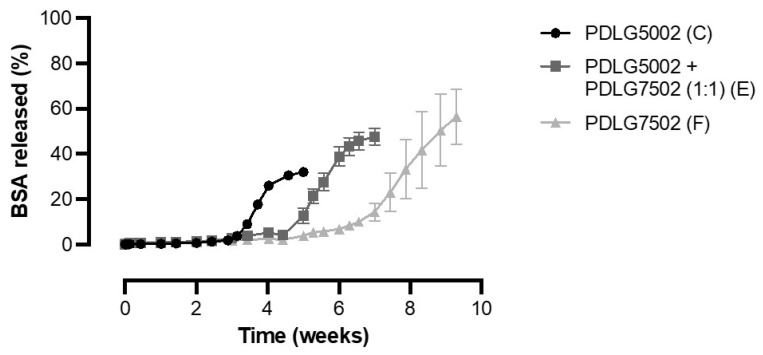
Cumulative in vitro release of BSA from core-shell microspheres composed of PLGA of different monomer ratios (*n* = 3).

BSA-containing core-shell microspheres with different shell compositions were produced to determine the influence of the PLGA monomer ratio on the in vitro release profile, as this monomer ratio greatly influences the degradation rate of PLGA. Figure 6 shows that all three formulations exhibited a delayed release profile without any BSA release during the lag phase. For formulation E, the release of BSA out of the microspheres indeed continued after the pulse, albeit at a decreased rate (data not shown). Moreover, there was a clear relationship between the monomer ratio of PLGA and the lag time, as the lag phase substantially increased with the increasing lactide content of the polymer. The lag time was approximately 3, 4.5, and 7 weeks for a PDLG5002, PDLG5002 + PDLG7502 (mass ratio 1:1), and PDLG7502 shell, respectively. A higher lactide content causes the hydrophilicity and thus the degradation rate to decrease [10,11,12]. In comparison, the lag time of the previously studied core-shell implants with a PDLG7502 shell was only 4.5 weeks [8]. A possible reason for this difference in lag time is that autocatalytic degradation starts after a few weeks of release. This autocatalytic degradation might play a bigger role in the large implants than in the thin-shelled microspheres [10,41]. A potential application of the produced core-shell microspheres is the current vaccine against SARS-CoV-2: for instance, the Pfizer-BioNTech vaccine that requires two doses given 3 weeks apart [42]. So far, only PDLG5002, PDLG7502, and a blend of both polymers were tested, but alternative monomer ratios could be used to tailor the in vitro release profile to the specific needs of different vaccines. Moreover, other studies with core-shell microspheres and implants have demonstrated that the lag time could be varied by altering the molecular weight of the polymer [29,43]. This opens many possibilities for the use of core-shell microspheres as single-injection vaccines.

## 4. Conclusions

This research demonstrates that monodisperse PLGA-based core-shell microspheres containing BSA can be produced using a microfluidic setup. In vitro release studies showed that after an adjustable lag time of 3 to 7 weeks, BSA released from the microspheres in a pulsatile manner, although the release was incomplete. This lag time was dependent on the monomer ratio of PLGA, with a higher lactide content causing a longer lag time. However, neither the shell thickness nor BSA loading had an influence on the release profile. These parenterally injectable delayed pulsatile release microspheres are a promising candidate for single-injection vaccine formulations when combined with a primer, as the lag time could be altered by varying the composition of the polymer shell. The primer dose could be included by injecting the core-shell microspheres together with an immediate-release formulation or a solution of the antigen. Moreover, even a second booster dose could be included by simply co-injecting core-shell microspheres with a different lag time. In this way, the release profiles can be tailored to the particular needs of a vaccine, which enables the use of core-shell microspheres for a wide variety of vaccines. Future research should focus on using alternative polymers that do not generate acidic degradation products to avoid incomplete protein release, and incorporating a therapeutically relevant vaccine.

## Figures and Tables

**Table 1:** Experimental parameters and settings of different bovine serum albumin (BSA)-loaded microsphere formulations.

Formulation	Model Compound	Polymer	Polymer Concentration (wt.%)	Flow Rates (W_1_–O–W_2_, µL/min)	Theoretical Loading (wt.%)
A	BSA	PDLG5002	10	0.58–7.8–50	9.3
B	BSA	PDLG5002	10	0.47–7.8–50	7.7
C	BSA	PDLG5002	10	0.40–7.8–50	6.7
D	BSA	PDLG5002	10	0.35–7.8–50	5.9
E	BSA	PDLG5002 + PDLG7502 (1:1)	7.5	0.28–7.8–30	6.4
F	BSA	PDLG7502	7.5	0.28–7.8–30	6.4
G	FITC-BSA	PDLG5002	10	0.20–5.4–40	1.0

**Table 2:** Characteristics of BSA-loaded core-shell microspheres of different grades of poly(DL-lactide-*co*-glycolide) (PLGA) and theoretical loading.

Formulation	Actual Loading (wt.%)	EE (%)	d50_particle_ (µm)	CV_particle_ (%)	d50_core_ (µm)	CV_core_ (%)	Shell Thickness (µm)
A	8.46 ^1^	90.56 ^1^	48.2 ± 1.8	3.8	41.3 ± 1.7	4.2	3.5 ± 0.6
B	6.91 ± 0.01	89.87 ± 0.17	43.4 ± 0.8	1.8	33.0 ± 1.2	3.6	5.2 ± 0.5
C	6.60 ± 0.06	98.48 ± 0.94	40.8 ± 1.2	2.9	29.5 ± 1.5	5.0	5.7 ± 0.6
D	1.37 ± 0.02	23.01 ± 0.41	38.1 ± 0.7	1.7	23.1 ± 0.6	2.6	7.4 ± 0.2
E	4.95 ± 0.43	77.74 ± 6.68	37.1 ± 2.8	7.6	28.5 ± 2.8	9.9	4.6 ± 1.2
F	5.73 ± 0.07	90.07 ± 1.03	46.0 ± 1.9	4.2	34.9 ± 3.9	11.2	6.3 ± 1.5
G	0.87 ± 0.04	86.72 ± 3.95	46.1 ± 2.8	6.1	35.0 ± 4.5	12.7	5.8 ± 0.9

^1^ For the determination of the actual loading, only one sample was analyzed so no standard deviation is given.

## Data Availability

Not applicable.

## Supplementary Materials

The following are available online at https://www.mdpi.com/article/10.3390/pharmaceutics13111854/s1, Video S1: video obtained with CLSM that visualizes the 3D structure of FITC–BSA fluorescence in PDLG5002-based core-shell microspheres.
